# HIV-reservoir size is not affected either by HCV coinfection or by direct acting antivirals (DAAs) therapy

**DOI:** 10.1038/s41598-022-08871-0

**Published:** 2022-03-24

**Authors:** Beatriz Álvarez, María A. Navarrete-Muñoz, Veronica Briz, Susana Olmedillas-López, Sara Nistal, Alfonso Cabello, Laura Prieto, Miguel Górgolas, Mariano García-Arranz, José M. Benito, Norma Rallón

**Affiliations:** 1 Hospital Universitario Fundación Jiménez Díaz, Madrid, Spain; 2 HIV and Viral Hepatitis Research Laboratory, Instituto de Investigación Sanitaria Fundación Jiménez Díaz, Universidad Autónoma de Madrid (IIS-FJD, UAM), Av. Reyes Católicos 2, 28040 Madrid, Spain; 3 Hospital Universitario Rey Juan Carlos, Móstoles, Spain; 4 National Center of Microbiology, Institute of Health Carlos III, Majadahonda, Spain; 5 New Therapy Group, Instituto de Investigación Sanitaria Fundación Jiménez Díaz, Universidad Autónoma de Madrid (IIS-FJD, UAM), Madrid, Spain

**Keywords:** Infectious diseases, Pathogenesis

## Abstract

The role of HCV on the HIV reservoir is controversial since the reduction on HIV-DNA levels after HCV eradication with IFNα/RBV treatment seems to be the result of drugs instead of HCV clearance. We assessed whether HCV eradication can decrease HIV-DNA content in HIV/HCV-coinfected patients treated with direct-acting antivirals, DAAs (IFNα/RBV-free regimens). Cell-associated HIV-DNA was measured by ddPCR in 25 HIV-monoinfected and 25 HIV/HCV-coinfected patients. There were no differences in HIV-DNA levels between groups neither at baseline nor at 12 weeks after DAAs treatment completion. Our results indicate that HCV does not appear to influence the HIV reservoir size and suggest the lack of an anti-HIV action for DAAs.

## Introduction

A relevant impact of HCV on very important aspects of the pathogenesis of HIV infection has been described, such as increased immune activation^1^, CD4 T cell apoptosis^2^ and T cell exhaustion^3^. However, other relevant aspects of HIV pathogenesis as HIV reservoir have been much less explored in the context of HIV/HCV coinfection. One study observed that HCV could boost HIV replication through the interaction with the HIV-LTR region^4^, which suggests a potential effect of HCV on HIV reservoir and therefore, a potential modulation of its size after HCV eradication. Some studies have explored the role of HCV on the HIV reservoir size, but with contradictory results^5–12^. In a previous cross-sectional study, we found increased levels of proviral HIV-DNA in HIV/HCV-coinfected patients compared to HIV-monoinfected^12^, but other cross-sectional studies have found opposite results also measuring proviral HIV-DNA, or 2 LTR circles^5^, or total HIV-DNA content^5,11^. Interestingly, in longitudinal studies, the evolution of HIV reservoir size after HCV eradication also remains a controversial issue^5–11^. The variation of HIV-DNA content after anti-HCV treatment with IFN-based regimens could be mediated by the immunomodulatory effects of IFNα and not necessarily to HCV clearance^5,6^. In fact, activity of IFNα against HIV has been described associated to upregulation of a group of host genes that can restrict viral replication in infected cells^13^. IFNα-induction of Natural Killer cells (NK) has been proposed as the possible mechanism underlying this anti-HIV activity of IFNα^14^. Given that the current scenario of Direct-Acting Antivirals (DAAs) regimens against HCV is free of IFNα, it is possible to test the direct effect of HCV eradication on the HIV reservoir. A few studies so far have tried to elucidate the role of DAAs in this issue^7–11^; however, no one have excluded patients with previous history of HCV therapy^7–11^; and most of them have evaluated the impact of DAAs but with RBV^7–10^.

The aim of this study was to evaluate the true role of the DAAs on HIV reservoir size modulation. We assessed whether the eradication of HCV could influence the HIV reservoir size in HIV/HCV-coinfected patients on successful combined Antiretroviral Therapy (cART) treated against HCV with DAAs IFNα/RBV-free regimens.

## Results

### Baseline characteristics of the study population

The main characteristics of patients at the time of inclusion in the study are shown in Table 1. There were no significant differences between HIV-monoinfected and HIV/HCV-coinfected groups in terms of age, gender, CD4 counts, plasma HIV-RNA load, risk group for HIV infection, time on combined antiretroviral therapy (cART) and time since HIV diagnosis. Respect to cART regimens, all HIV/HCV-coinfected patients received a regimen based on a nucleoside analog reverse-transcriptase inhibitor (NRTI) and an integrase inhibitor (INI) (100%), while HIV-monoinfected were treated with a backbone of NRTI associated with an INI in 56% of patients, with a non-nucleoside reverse-transcriptase inhibitor (NNRTI) in 36% of patients, and with a protease inhibitor (PI) in 8% of patients. Regarding anti-HCV treatment, the majority of patients were treated with the combination of Elbasvir/Grazoprevir (16 out of 25), followed by the combination of Sofosbuvir/Ledipasvir (8 out of 25) and only 1 patient treated with the combination of Ombitasvir/Paritaprevir/Ritonavir/Dasabuvir. There were no significant differences in the prevalence of different comorbidities between the two groups of patients.

**Table 1:** Characteristics of patients at the time of inclusion in the study.

Characteristic	Study group		p-value
	HIV-monoinfected patients	HCV/HIV-coinfected patients	
n	25	25	–
Age (years)	48 [41–54]	44 [38–47]	0.106
Male (%)	88	100	0.074
HIV Viral load (copies/mL)	< 50	< 50	NA
CD4 counts (cells/µL)	816 [604–991]	735 [577–902]	0.313
Time since HIV diagnosis (years)	9 [6–13]	7 [2–10]	0.072
Time on cART (years)	5 [3–7]	4 [2–9]	0.632
**Type of ART regimens**			0,001
NRTI + NNRTI	36	0	
NRTI + INI	56	100	
NRTI + PI	8	0	
**Risk group for HIV infection**			0.312
Sexual transmission (%)	100	96	
Parenteral transmission (%)	0	4	
Prevalence of comorbidities (%)	44	40	0.774
**Type of comorbidities (%)**			
AIDS related events	8	20	0.221
Cardiovascular	8	20	0.221
Metabolic	16	8	0.384
Renal	0	4	0.312
Osteoporosis	20	4	0.082
Cancer	0	4	0.312

Data are expressed as median [IQR], except sex and risk group for HIV infection, expressed as %; p-value: comparison between HIV-monoinfected and HCV/HIV-coinfected groups (U-Mann–Whitney test), except sex, type of ART regimens, risk group for HIV infection and comorbidities (evaluated with χ^2^ test).

*NA* not apply, *NRTI* nucleoside analog reverse-transcriptase inhibitor, *NNRTI* non-nucleoside reverse-transcriptase inhibitor, *INI* integrase inhibitor, *PI* protease inhibitor.

### HIV reservoir size is independent of HCV coinfection and remains stable after HCV eradication with DAAs treatment

**Figure 1:**
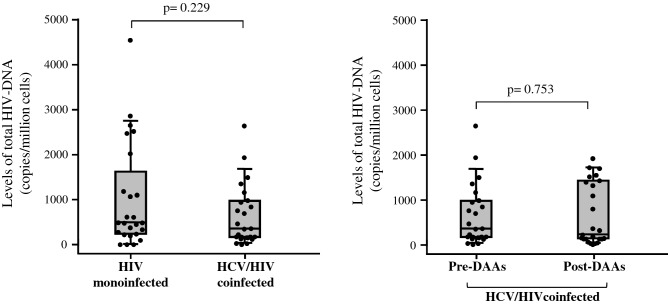
Levels of total HIV-DNA content in purified CD4 + T cells. Whisker Box-plots showing the levels of total HIV-DNA content in purified CD4 + T cells from HIV-monoinfected and in HCV/HIV-coinfected patients (left graph); and levels of total HIV-DNA content in CD4 + T cells from HCV/HIV-coinfected patients before and after HCV eradication with DAAs-based treatment (right graph). p-values for inter and intra-group comparisons (Mann–Whitney U test and Wilcoxon signed-rank test, respectively) are shown.

To investigate the potential impact of HCV coinfection and HCV eradication on HIV reservoir size, we performed both a cross-sectional and a longitudinal analysis of total HIV-DNA content in purified CD4^+^ T cells from 50 cART-treated patients: 25 HIV-monoinfected patients and 25 HIV/HCV-coinfected patients in whom HCV was eradicated with DAAs-based anti-HCV treatment. HIV-DNA content was quantified at a single timepoint in all patients and at 12 weeks after completion of HCV treatment in the group of HIV/HCV-coinfected patients. We first compared basal levels of total HIV-DNA (median [IQR] copies/million cells) between HIV/HCV-coinfected and HIV-monoinfected patients and did not found significant differences (348[156–962] and 485[229–1605] copies/million cells respectively; p = 0.229) (Fig. 1). A Generalized Linear Model (GLM) analysis confirmed this lack of association between HIV-DNA level and presence of HCV coinfection after adjusting by potential confounding variables (time since HIV diagnosis, time on cART and CD4 counts) (p = 0.095, data not shown). We then compared total HIV-DNA content in HIV/HCV-coinfected patients before and 12 weeks after DAAs treatment completion to observe potential changes in HIV reservoir size as a consequence of HCV eradication. Again, we did not observe significant differences in the levels of total HIV-DNA content (348[156–962] and 218[127–1405] copies/million cells before and after anti-HCV treatment respectively; p = 0.753) (Fig. 1). In addition, no association between HIV-DNA level and HCV eradication by DAAs was observed after adjusting by the same potential confounding variables (time since HIV diagnosis, time on cART and CD4 counts) with a GLM analysis (p = 0.581, data not shown). We also evaluated if the type of DAAs combination could influence in the HIV reservoir size and found no association since the level of HIV-DNA 12 weeks after anti-HCV treatment was similar between patients treated with Elbasvir/Grazoprevir combination and those treated with Sofosbuvir/Ledipasvir combination (285[168–895] and 245[121–1225] HIV-DNA copies/million cells respectively; p = 0.713).

## Discussion

In the present study we have assessed the impact of HCV coinfection on HIV reservoir size using two different study designs: a cross-sectional design to test the effect of active HCV infection on HIV reservoir size; and a longitudinal design to analyze the evolution of HIV reservoir size after HCV eradication. Moreover, given the direct antiviral effect of DAAs, our study has analyzed the impact of HCV eradication on HIV reservoir size without the bias of an immunomodulatory action as observed with previous IFNα and/or ribavirin-containing regimens. Also, it has to be noted that, in contrast to most previous studies, which measured HIV reservoir in total peripheral blood mononuclear cells (PBMCs), in our study the HIV reservoir size was measured in purified CD4^+^ T cells and using a highly accurate and reproducible methodology in the context of a low level of detection (as is the case of patients on successful cART with undetectable levels of HIV viral load). Measuring the HIV reservoir in total PBMCs is a source of bias when estimating the concentration of latently infected cells. PBMCs consist of a mixture of various cell types, not all of which are susceptible to HIV infection and to the establishment of a reservoir.

Firstly, we did not find significant differences in the level of HIV-DNA content in purified CD4^+^ T cells when comparing HIV/HCV-coinfected and HIV-monoinfected patients on successful cART, even after adjusting for relevant confounding variables. We previously found increased levels of proviral HIV-DNA in HIV/HCV-coinfected patients compared to HIV monoinfected^12^. This discrepancy could be found in the different cohorts of patients included; the different levels of inflammation and liver fibrosis in the studied patients; the different cell populations used to assess the HIV reservoir; and the different methodologies used to measure the HIV reservoir. Interestingly, our current results (using a very homogeneous cohort of HIV/HCV-coinfected and HIV monoinfected patients with paired parameters and with strict selection criteria; in addition, the use of a highly precise and reproducible methodology to measure the HIV reservoir, as well as the use of a highly purified population of CD4^+^ T cell) are in agreement with previous^5^ and more recent^11^ cross-sectional studies in different cohorts of HIV/HCV-coinfected and HIV-monoinfected patients under cART^5,11^. Thus, the results of our current cross-sectional study demonstrate that presence of HCV coinfection do not significantly impact on the HIV reservoir size in HIV patients with successful cART.

Secondly, the results of our longitudinal study revealed that HIV-DNA content did not significantly change after HCV eradication, what reinforces the conclusion of our cross-sectional study. The results of our longitudinal study are also in agreement with most previous studies analyzing the evolution of HIV-DNA content after HCV eradication with anti-HCV therapies based on DAAs^7,8,10,11^. In contrast, one study found a significant decrease of HIV-DNA level in HIV/HCV-coinfected patients after HCV eradication^9^. However, most patients in that study had past history of HCV treatment, and now were treated using DAAs plus Ribavirin. Also, the content of HIV-DNA was evaluated in total PBMCs not in CD4^+^ T cells. More important, the same authors in a later article with a different cohort of patients contradict their own results, with new findings regarding no significant differences in HIV-DNA levels in HIV/HCV-coinfected patients after HCV eradication but this time using DAAs regimens against HCV free of RBV with no reported history of HCV-pretreated^11^.

Finally, there are some caveats that deserve comment: we analyzed total HIV-DNA and thus we could not rule out a potential effect of HCV on other parameters such as integrated HIV-DNA or, more importantly, replication-competent reservoir. However, the majority of HIV-DNA in long-term cART-treated patients is integrated^15^, and both integrated and total HIV-DNA predict ex vivo viral outgrowth^16^ suggesting that total HIV-DNA is a good surrogate of the size of replication-competent reservoir. We evaluated the evolution of HIV-DNA in the HIV-HCV coinfected population after 12 weeks post-HCV treatment; and thus, we cannot rule out the possibility of variation on HIV-DNA levels in the long-term after HCV eradication. Interestingly, a very recent study evaluating total; integrated; and 2LTR HIV-DNA in purified CD4^+^ T cells found that all different forms of HIV-DNA measured remained stable from basal time to 12 months after HCV eradication with DAAs^10^.

It has to be noted that, in contrast to most previous studies^6–10,12^, our study included a cross-sectional and a longitudinal design and both yielded the same results, what reinforces the overall conclusion. In summary, the results of our study do not support a role for HCV on promoting changes in HIV reservoir size. Moreover, our findings highlight that HCV eradication with DAAs therapy does not affect the size of HIV reservoir. These findings may have clinical relevance in future clinical trials aimed at diminishing the HIV reservoir size.

## Materials and methods

### Study design

This is a retrospective longitudinal study conducted in two groups of cART-suppressed chronic HIV-infected patients maintaining undetectable plasma viral load (pVL): 25 HIV-monoinfected patients and 25 HIV/HCV-coinfected patients, who were naive for anti-HCV therapy at the time of their inclusion in the study, regularly followed at outpatient clinic of Hospital Fundación Jiménez Díaz in Madrid. The period of study spanned from 2016 to 2018. In the HIV/HCV-coinfected patients, samples of peripheral blood mononuclear cells (PBMCs) prior to the start of treatment with DAAs and at 12 weeks after its completion (when sustained virological response -SVR- is assesed) were collected. In the HIV-monoinfected group a single cellular sample was collected from each patient. The study protocol was approved by the Ethical review board of Instituto de Investigación Sanitaria-Fundación Jiménez Díaz, Madrid, Spain. Each participating individual signed an informed consent form. All experiments were performed in accordance with relevant guidelines and regulations.

### Isolation of total CD4^+^ T cells

CD4^+^ T cells were isolated by immunomagnetic enrichment from 20 million of PBMCs using an immune-magnetic separation technique (MACS microbeads system, Miltenyi Biotec, Spain) according to the manufacturer’s instructions. CD4^+^ T cells were obtained by negative selection with CD4^+^ T Cell Isolation Kit, that includes a cocktail of monoclonal antibodies against CD8, CD14, CD15, CD16, CD19, CD36, CD56, CD123, TCR γ/δ, and CD235a (Miltenyi Biotec, Spain). The purity of the CD4^+^ T cells was always > 90% as assessed by flow cytometry (median [IQR] purity: 94% [91–96%]).

### Quantification of HIV reservoir size

Total DNA from purified CD4^+^ T cells of each patient was extracted using QIAamp DNA-MiniKit (Qiagen, USA) following the manufacturer’s instructions. HIV reservoir size was measured as total cell-associated HIV-DNA by ultrasensitive digital-droplet PCR (ddPCR), a technique with greater precision and reproducibility in the context of a low level of detection (as is the case of our study with patients on successful cART) when compared with techniques that measure integrated HIV-DNA^17^. We used ddPCR Supermix for probes (Bio-Rad, USA) according to manufacturer’s recommendations. We employed primers/probe sets annealing to the GAG conserved region of HIV-1 genome (F: 5’-AGTTGGAGGACATCAAGCAGCCAT GC AAAT-3’, R: 5’-TGCTATGTCAGTTCCCCTTGGTTCTCT-3’; Probe: 5’-GACCATCAATGAGGAAGCTGCAGAATGGGAT-3’) (Bio-Rad, USA). A commercially available set of primers/probe for RPP30 cellular gene (Bio-Rad, USA) was used to normalize sample input. Data was acquired and analyzed using a QX100™ droplet reader (Bio-Rad, USA) and the QuantaSoft v.1.6 software (Bio-Rad, USA). DNA from HIV-negative subjects were included in each plate as negative controls and to set the positive/negative threshold for ddPCR analysis.

### Statistical analysis

Inter and intra-group comparisons were performed using non-parametric tests (Mann–Whitney U test and Wilcoxon signed-rank test respectively), and the potential effect of different confounding variables was checked by analysis of variance in a generalized linear model (GLM) framework. All statistical analyses were performed using the SPSS software version 15 (SPSS Inc., Chicago, IL, USA). All p-values were two-tailed, and were considered as significant only when lower than 0.05.
